# Using Ground Penetrating Radar to Reveal Hidden Archaeology: The Case Study of the Württemberg-Stambol Gate in Belgrade (Serbia)

**DOI:** 10.3390/s20030607

**Published:** 2020-01-22

**Authors:** Aleksandar Ristić, Miro Govedarica, Lara Pajewski, Milan Vrtunski, Željko Bugarinović

**Affiliations:** 1 Faculty of Technical Sciences, University of Novi Sad, Trg Dositeja Obradovica 6, 21000 Novi Sad, Serbia; aristic@uns.ac.rs (A.R.); miro@uns.ac.rs (M.G.); milanv@uns.ac.rs (M.V.); zeljkob@uns.ac.rs (Ž.B.); 2 Department of Information Engineering, Electronics and Telecommunications, Sapienza University of Rome, via Eudossiana 18, 00184 Rome, Italy

**Keywords:** ground penetrating radar (GPR), urban archaeology, non-destructive testing (NDT), three-dimensional analysis

## Abstract

This paper presents the results of a research study where ground penetrating radar (GPR) was successfully used to reveal the remains of the Württemberg-Stambol Gate in the subsurface of Republic Square, in Belgrade, Serbia. GPR investigations were carried out in the context of renovation works in the square, which involved rearranging traffic control, expanding the pedestrian zone, renewing the surface layer, and valorising existing archaeological structures. The presence of the gate remains was suggested by historical documents and information from previous restoration works. A pulsed radar unit was used for the survey, with antennas having 200- and 400-MHz central frequencies. Data were recorded over a grid and two three-dimensional models were built, one for each set of antennas. The grid was the same for both sets of antennas, therefore the two models could be compared. Several horizontal cross sections of the models were plotted, corresponding to different depths; these images were carefully examined and interpreted, paying particular attention to signatures that could originate from the sought archaeological structures. Reflections coming from the gate remains were identified in both models, in the same region of the survey area and at the same depth; the geometry, size, and layout of the gate columns, as well as of other construction elements belonging to the gate, were determined with very good accuracy. Based on the GPR findings, archaeological excavation works were carried out in the region where the foundation remains were estimated to be. The presence of the remains was confirmed, with various columns and side walls. This case study demonstrates and further corroborates the effectiveness and reliability of GPR for the non-invasive prospection of archaeological structures hidden in the heterogeneous subsurface of urban environments. In the opinion of the authors, GPR should be incorporated as a routine field procedure in construction and renovation projects involving historical cities.

## 1. Introduction

Ground penetrating radar (GPR) is a sensing device that uses low-power electromagnetic waves to produce high-resolution images of the subsurface and interior of objects [1,2]. A GPR typically transmits short electromagnetic pulses of energy into the structure under test, within the 100 MHz–4 GHz frequency range. When the electromagnetic waves emitted by the radar encounter a buried object or, more in general, a discontinuity of electric and magnetic properties, what happens is that reflection, refraction, transmission and scattering phenomena occur, hence part of the energy is echoed back to the GPR. By exploiting advanced data processing and imaging techniques [1,3], the electromagnetic signals recorded by the radar can be transformed into useful two-dimensional (2D) or three-dimensional (3D) images, which basically enables seeing into a structure that is opaque to the human eye.

GPR has a plethora of different applications and plays an important role in the management and preservation of our cultural heritage [4,5]. Several papers are available in the scientific literature, which illustrate the use of GPR to discover and map buried archaeological artefacts, to inspect ancient buildings and monuments, bridges, columns and statues, to investigate frescoes, mosaics and decorations, and to analyse the internal conditions of various other objects of historical value [6,7,8,9,10,11,12,13,14,15,16,17]. 

As far as GPR archaeological prospection is concerned, GPR has been introduced into archaeology in the 1970s: in [18], the surveys conducted at Chaco Canyon, New Mexico, in 1974–1976 [19] are considered as the first, partly successful GPR archaeological study. GPR archaeological surveys started as small-scale approaches to sense buried features and estimate their main geometric and physical properties. In the last fifteen years, thanks to advances in technology, the sensitivity and resolving power of GPR systems has significantly increased; moreover, the advent of multi-channel radar systems equipped with antenna arrays has permitted an increase in survey efficiency and spatial sampling resolution [20]. The availability of software for advanced data processing [21] and full-wave electromagnetic simulation [22] has been decisive for a more sophisticated application of the GPR technique, too. In [23], the state-of-the-art in large-scale high-resolution archaeological GPR prospection is presented, covering hardware and software technology and fieldwork methodology, as well as modern processing approaches and interpretation of huge data sets. 

An important task that can be tackled by the GPR technique is the preventive detection of ancient structures at locations of current or future construction works; this situation is quite common in historical urban areas where, due to the city development, various renovation works may be necessary in old districts [24,25,26]. 

Very often GPR is combined with other survey methods, such as remote sensing (e.g., hyperspectral images and aerial laser scanning), geophysical techniques (e.g., electrical resistivity tomography and magnetic gradiometry), and other non-invasive approaches [27,28,29].

GPR is not straightforward to use and does not give its best in the hands of an unskilled operator: its successful application requires proper training and initially also supervision by an expert, both in using the radar to collect the measurements and in interpreting the data. The results of archaeological GPR investigations strongly depend not only on the skills of the operator but on a series of environmental factors too: the water content and mineral composition of the soil; the size, shape, burial depth and conservation state of the sought remains; the topography of the survey area; and, the heterogeneity of the environment, namely the presence of targets other than the sought ones, such as stones, debris, roots, utilities, and more.

While uniform sandy soils offer ideal conditions for archaeological GPR prospection, historical cities are extremely challenging environments. Buildings, walls, streetlamps, road signs, trash cans, curb stones, traffic islands, manholes and gully covers, trees and vehicles are examples of scattering objects typically found over the urban surface, which cause the presence of disturbing anomalies in the radar data. Variations in the surface cover (roadway and sidewalk asphalt layers having different properties, cobblestones, paving, soil, gravel, grass) generate differences in the electric-field amplitudes received by the GPR. Additionally, in the shallow subsurface there usually are many pipes and cables made of different materials and laid along different directions, rubble and debris; there can also be tree roots, backfilled excavation trenches and pits, cellars and cavities, wells and tunnels, graves, foundation walls of former constructions, and more.

Different data acquisition strategies are possible when using a GPR. The simplest method is the acquisition of single traces (A-Scans) in a series of specific points: in archaeological surveys, this approach is adopted when the irregularity of the ground does not allow moving the radar antennas along a line. Common-offset data acquisition along isolated lines provides more information about the subsurface, as it yields 2D vertical radar images of the ground (B-Scans); in archaeological surveys, this method is normally used in narrow areas. The optimal method is the common-offset data acquisition along several lines, over a regular grid; this approach allows to obtain a 3D radar image (C-Scan) of the subsurface and horizontal radar maps at different depths (slices), which are extremely useful for an accurate interpretation of the data and to identify the anomalies of interest. In case of strongly uneven terrain and presence of obstacles, it is also possible to obtain interpolated radar images from data recorded over irregular grids. 

In 2013–2017, European Cooperation in Science and Technology (COST) Action TU1208 “Civil engineering applications of Ground Penetrating Radar” contributed remarkably to the advancement of GPR research, not only in the civil-engineering field, but also in the cultural heritage area of application [30,31]. The Action helped to overcome the fragmentation of funding in the context of Horizon2020, by providing coordination, networking and training opportunities to more than three hundreds GPR researchers and innovators [32,33]; it also helped to raise awareness about GPR capabilities and applications in the European continent, and to establish a better dialogue between scientists, stakeholders and end-users of GPR research through a series of science communication initiatives some of which were dedicated to the use of GPR in archaeological prospection [34]. 

This paper presents the results of a new case study recently carried out in Belgrade, Serbia, which demonstrates an advanced use of GPR to obtain reliable and useful information concerning archaeological remains hidden in the heterogeneous and complex subsurface of a historical city. A commercial pulsed radar unit was used for this survey, with two sets of antennas having 200 MHz and 400 MHz central frequencies. Data were recorded over a 2D grid and two 3D models were created, one for each set of antennas. The grid was the same for both sets of antennas, therefore the two models could be compared. Several horizontal cross sections of the models were extracted, corresponding to different depths; these images were examined and interpreted, paying special attention to signatures that could originate from the sought archaeological structures. Based on the GPR findings, excavation works were carried out in the region where the archaeological remains were estimated to be. Preliminary results of this research study were presented at the 2019 European Geosciences Union General Assembly (EGU GA) [35].

## 2. The Survey Area and Sought Archaeological Remains

**Figure 1:**
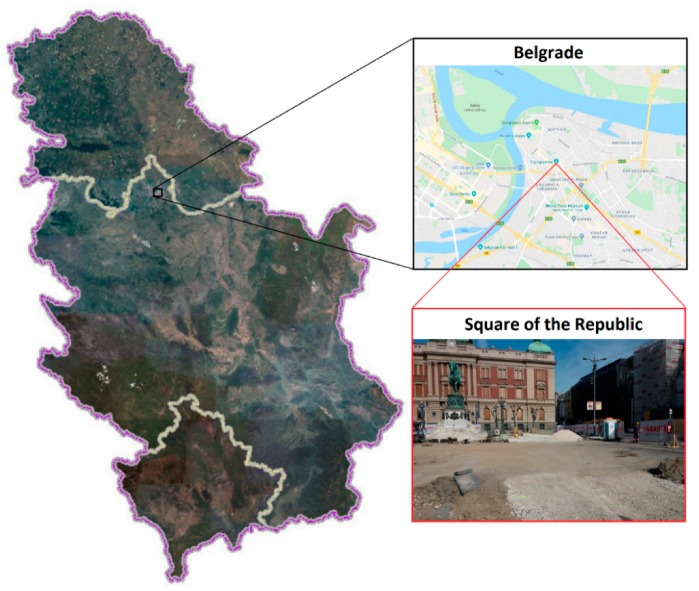
Geographical location of the GPR survey. The photo of Square of the Republic was taken at the time of the survey.

The survey was carried out in Square of the Republic, located in Belgrade, Serbia, which is in the very centre of the city (Figure 1). The square hosts some of the city’s important public buildings, including the National Museum, the National Theatre, and the statue of Prince Michael. The goal of the GPR survey was to find and localize the buried remains of the Stambol Gate foundations in the subsurface of the Square of the Republic.

The centre of Belgrade is a complex urban area where the development of human society went through several stages, in different epochs of history. Some events that happened during the 18th century are of particular interest for the GPR study presented in this paper. In that period, after the war between the Austrian and Ottoman Empire, Belgrade was run by Austrians. A typical baroque gate was built in 1725 as part of the city defense system and it was named after Carl Alexander von Württemberg, who governed the city and the Kingdom of Serbia from 1720 until 1733. Subsequently, the Kingdom of Serbia was governed by Karl Christoph von Schmettau (1733–1738), by George Oliver de Wallis (1738–1739), and between 1739 and 1806 Austrians and Ottomans switched in running the city of Belgrade several times. After the Austrian army was defeated in the battle near Grocka, in 1739, Austria agreed to sign a truce. Austrians insisted on demolishing all objects and fortifications built in the period of their rule. In June 1740 an agreement was signed, and many constructions were demolished, including the Württemberg Gate. From the available descriptions, it is known that Württemberg Gate was large and monumental, but there are no surviving illustrations, so the exact appearance of the gate is unknown. 

In 1740, the Ottomans decided to build a new gate in Belgrade, inspired by the Württemberg Gate and in the same position where the demolished gate was previously located. The new gate was the largest of all city gates at the time and it was considered the most beautiful. It was named Stambol Gate because it was located at the starting point of the Tsarigrad Road, which linked Belgrade with Constantinople (Istanbul).

The Stambol Gate was made of dressed stone and bricks, on a rectangular base. It had rooms for housing the sentinels and three entry points: a large central one, for the carriages, and two smaller ones on the sides, for the pedestrians. However, like the Württemberg Gate, there are no reliable detailed descriptions of the Stambol Gate and its exact appearance is unknown. Some drawings can be found in the literature, but they are just artistic illustrations and can mislead the interpretation of GPR data. 

Stambol Gate gate became notorious as the place in front of which the Turks executed the rayah, their non-Muslim subjects. It was also the place where during the attack on Belgrade in 1806 in the First Serbian Uprising, one of the leading Serbian military commanders, Vasa Čarapić, was fatally wounded. The gate continued to play a strategic role until the 19th century. After 1815, when Serbia was granted autonomy, Ottoman guards were placed at the gate to control the entry into the fortress. Finally, the gate was demolished in 1866, on the orders of Prince Michael, since it was a symbol of Serbian sufferings during the Ottoman Empire. The stones from the gate were re-used for the construction of the surrounding houses and for the building of the National Theatre. 

When the Square of the Republic was renovated in 1928–1929, the remains of the Württemberg-Stambol Gate’s foundations were discovered beneath the pavement, however it was not recorded whether the remains were covered again or removed. In 1949, during further renovation works in the square, the remains were discovered again: this time, geodesists surveyed them, and the resulting maps were included in the cadastral plan of Belgrade.

In 2019, major construction works were planned and executed in Square of the Republic, which included rearranging traffic control, expanding the pedestrian zone, renewing the paving of the square, and valorising the presence of buried archaeological features. It was decided to incorporate a GPR survey in the construction works, to preliminarily check and accurately identify the position and size of the remains of the Württemberg-Stambol Gate’s foundations. 

## 3. Methodology

### 3.1. Survey Planning and Data Acquisition Strategy

**Figure 2:**
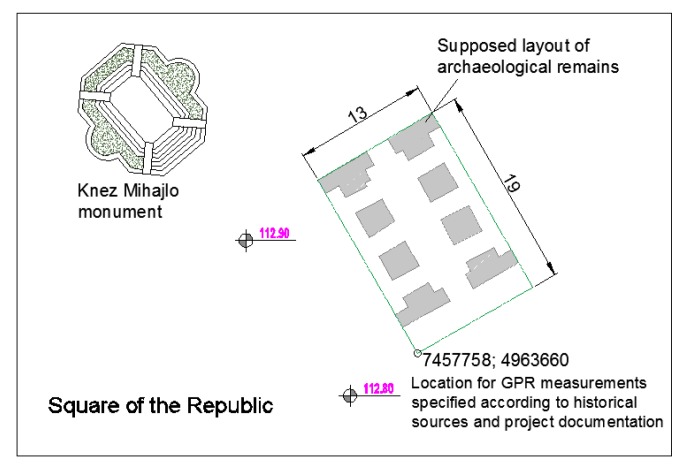
Survey location determination: extraction of the supposed location of the foundation remains based on geodetic archives (state plane coordinates).

The survey area was almost 250 m^2^ wide and it was defined based on the available documents and information [36,37,38], including the geodetic maps mentioned in Section 2 (Figure 2).

According to the presumed size and burial depth of the gate, the most appropriate GPR equipment was chosen and suitable survey settings were defined, such as the number of radargrams to be recorded, the spatial sampling, the time window, and more.

In particular, all measurements were carried out by using a pulsed GPR system manufactured by Geophysical Survey Systems, Inc. (SIR 3000, GSSI, Nashua, NH, USA) mounted on an in-house cart and equipped with two sets of ground-coupled shielded antennas having 200 MHz and 400 MHz central frequencies, respectively. The SIR 3000 control unit was released in 2002 and is currently not for sale, but the existing units still are very much used; this radar is especially suitable for concrete-structure and bridge-deck inspection, utility location, geological investigations, archaeological surveys, forensics, and mining. The 200 MHz antennas were chosen to achieve a scanning depth of 4 m. These antennas are ideally suited for geotechnical and environmental applications, as well as archaeological investigations; depending on the subsurface properties, they have a depth range of 0 to 9 m. The 400 MHz antennas were chosen to obtain a better resolution in the shallow sub-surface; in the region of the survey, their expected scanning depth was 2 m. These antennas are ideally suited for detection and mapping of utility pipes, void detection, tunnel voids, and archaeological applications; depending on the subsurface properties, they have a depth range of 0 to 4 m.

**Figure 3:**
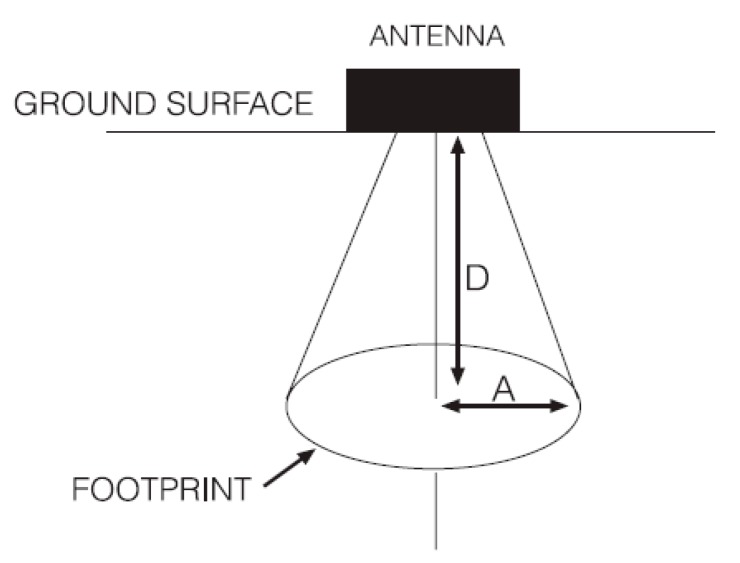
Antenna footprint (A) calculation, geometry of the problem.

Due to the expected geometrical properties of the sought structures and considering the size of the survey area, it was noticed that taking measurements over a 2D grid and creating 3D models of the subsurface out of GPR data was the best way to perform the investigation. A 19 m × 13 m grid was designed. The long-established common-offset acquisition method was chosen, where the transmitting and receiving antennas are moved together along the acquisition line. The preparation of radargram acquisition comprised the following phases:

(1) Marking of the 19 m × 13 m zone that included the region of interest. This step was done by using a Global Navigation Satellite System (GNSS) device and terrestrial stake-out of the points with coordinates read from cadastral plans [38].
(2) Estimation of the average propagation velocity of electromagnetic waves (or the average relative permittivity of the soil) in the region of the survey. Such information is used to determine the most appropriate distance between adjacent acquisition lines and it is crucial to set the time window and calibrate the GPR data, i.e., to transform time into distances. The estimation of the average wave velocity was accomplished by scanning underground utilities at known depth [39,40]. Since there were several manholes in the zone, with utilities 1 to 2 m deep, this procedure was repeated many times to find a representative value of the velocity. The estimated value was *v* = 0.106 [m/ns], which corresponds to a relative permittivity ε_R_ = 8. Based on these observations, the average wavelength λ in the soil was calculated for both the antenna central frequencies 400 MHz and 200 MHz (see Table 1).
(3) Verifying that the chosen vertical scanning resolution satisfied Nyquist sampling criterion. According to Nyquist sampling criterion, the vertical scanning resolution has to be Δy ≤ 0.25λ [41,42]. When using the 400 MHz antennas, the time window was chosen by taking into account the estimated value of *v* and to achieve a scanning depth of 2 m; with 512 samples per trace, it was easy to check that Nyquist criterion was satisfied (2 m/512 = 0.039 m < 0.066 m, Table 1). When using the 200 MHz antennas, the desired scanning depth was 4 m and the vertical resolution was again 512 samples/scan, hence Nyquist condition was satisfied in this case too (4 m/512 = 0.0078 m < 0.1325 m, Table 1).
(4) Choosing an appropriate distance between adjacent acquisition lines (profile spacing, PS). PS plays a decisive role in GPR archaeological prospection [43]. A denser profile spacing yields better horizontal resolution, higher quality of horizontal slice images and easier data interpretation—but is time consuming, hence expensive. Obviously, it is not realistic to acquire infinitely dense GPR profiles and in practice it is always necessary to balance between survey resolution and its cost; interpolation techniques are widely used to fill data gaps between adjacent profiles [43]. In some cases, different PS values may be chosen along different grid axes. All things considered, a good value for PS can be chosen in various ways, as summarized in the following:
  (a) Full resolution scanning [41]–this approach provides a horizontal resolution that eliminates the need for interpolation. Results obtained using this procedure are the best, especially for archaeological prospection, but they are very time consuming. The approach requires that the chosen PS satisfies Nyquist sampling criterion, i.e., PS ≤ 0.25λ. In the case study presented herein, PS should therefore be about 7 cm for the 400 MHz antennas and about 14 cm for the 200 MHz antennas. If higher frequency values are used in this calculation, taking into account that the emitted pulse has a wide spectrum, PS becomes even smaller. Such a dense profiling is very difficult to obtain in field conditions: grid marking on the ground would take too much time and be tedious, as well as the acquisition itself. This approach is applicable only in case of ideal ground surface and without time constraints for the acquisition.
  (b) Definition of PS based on the expected size of the sought targets and distance between adjacent targets–This is the simplest and most common approach, wherein the chosen PS is no larger than the expected size of the sought objects and no larger than the distance between adjacent objects [44,45,46]. In the case study presented in this paper this approach was adopted and PS = 0.5 m was chosen. On the 19 m × 13 m grid, 39 + 27 = 66 radargrams were therefore collected, whereas the application of the full resolution scanning approach would have required the collection 186 + 272 = 458 radargrams with the 400 MHz antennas, and 98 + 136 = 229 radargrams with the 200 MHz antennas.
  (c) Definition of PS based on the analysis of a *f-k* (frequency-wavenumber) plot of a densely sampled, representative radargram. This is an often-practiced graphical procedure that has to be executed in the field by the GPR operator, taking into account also the maximum radiation angle of the GPR antenna over the specific soil where the survey is carried out. This approach was not used in the present case study and it is not discussed further in this paper, but a detailed explanation can be found in [41].
  (d) Calculation of PS based on the antenna footprint (Figure 3)–In this method, the calculation of the antenna footprint at a given depth is used to choose an appropriate spatial resolution. In particular, the radius of the antenna footprint is the minimum value of PS that guarantees full coverage of the area of interest at a given depth. Different equations can be used to estimate the antenna footprint, based on different empirical definitions of the Fresnel distance. According to [5]:
    (1) $$A=\frac{\lambda }{4}+\frac{D}{\sqrt{\varepsilon_{R}+1}}$$
    and according to [44]:
    (2) $$A=\sqrt{\left(\frac{v^{2}}{16f^{2}}+\frac{vD}{2f}\right)}$$
    where *A* is the radius of the antenna footprint, λ is again the wavelength at the central frequency *f* of the used antennas, *v* is the wave velocity, *D* is the depth from the ground surface to the reflection surface where the antenna footprint is calculated, and ε_R_ is the average relative dielectric permittivity of the material from the ground surface to the depth *D*. For the antennas used in this paper and given conditions in the field, the resulting values of *A* when *D* = 1 m and *D* = 2 m are reported in Table 1. The expected depth of the sought targets was between 0.5 m and 1.8 m, and the values of *A* at 1 m and 2 m, according to equations (1) and (2), were between 0.37 m and 0.8 m. These results confirm that PS = 0.5 m is a reasonable value.
  (e) In [44], it is suggested to use PS < 4λ, which in the present case study yields a minimum PS of 1.06 m and 2.12 m with 400 MHz and 200 MHz antennas, respectively. This further confirms that PS = 0.5 m was an appropriate choice for both sets of antennas.

**Figure 4:**
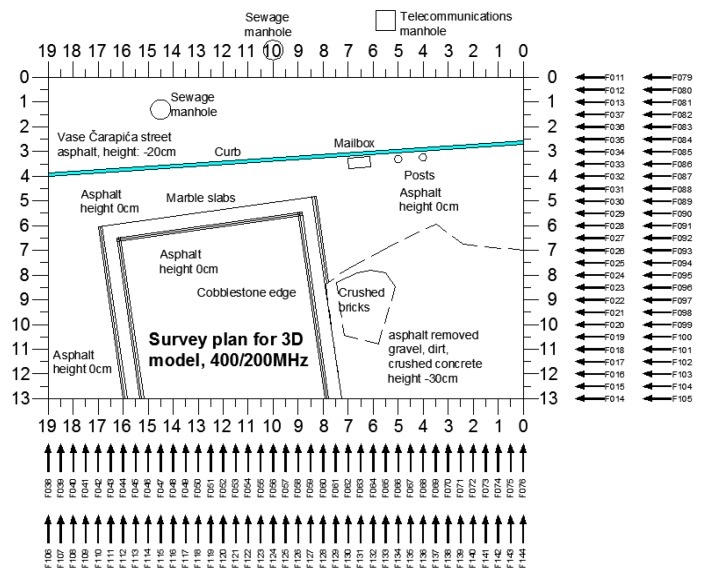
Survey plan showing radargram position and orientation for both antennas.

**Figure 5:**
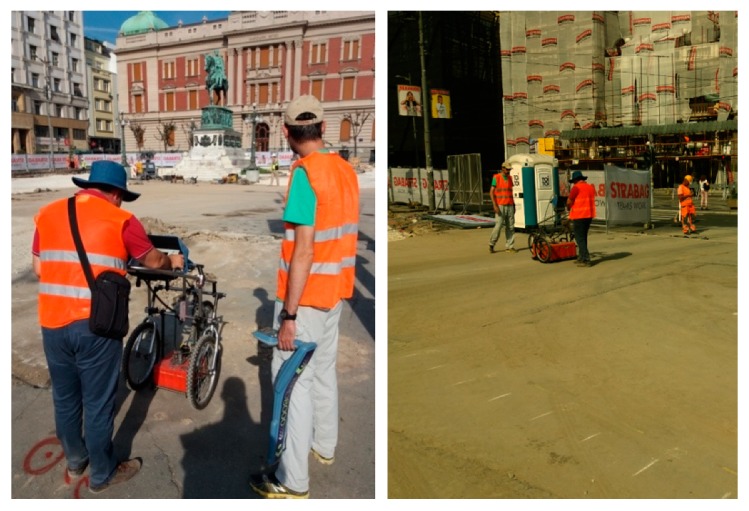
GPR data acquisition in Square of the Republic.

The GPR scanning plan, with marks of radargrams recorded using both sets of antennas, is shown in Figure 4; the radargrams were recorded using a fixed starting line per axis, to minimize the profile georeferencing error in the 3D models. Since data were to be used to create 3D models, all GPR settings were the same during all acquisitions with a given set of antennas. Figure 5 consists of photos taken during the measurements, with both antennas. The survey was planned and executed taking into account the practical recommendations given in [47,48] for a safe use of GPR during near-surface geophysical archaeological prospection.

**Figure 6:**
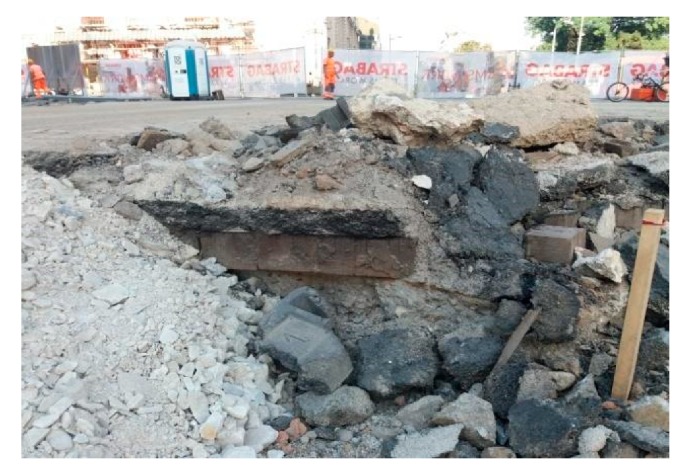
Photo taken at Square of the Republic during the GPR survey, showing the region where the surface layer had already been removed.

Several obstacles were present in the survey area, such as curbs and street lighting posts. In a region of the surveyed zone, the surface layer had already been removed down to a 0.5 m depth, making the acquisition of a few profiles more difficult (Figure 6). In the first 60 cm of the subsurface, the following layers were present: cobble/marble plate, about 8 cm thick; base layer, about 8 cm thick; asphalt, about 6 cm thick; wooden cubes, about 11 cm thick; concrete, about 20 cm thick; these layers were followed by the soil.

**Figure 7:**
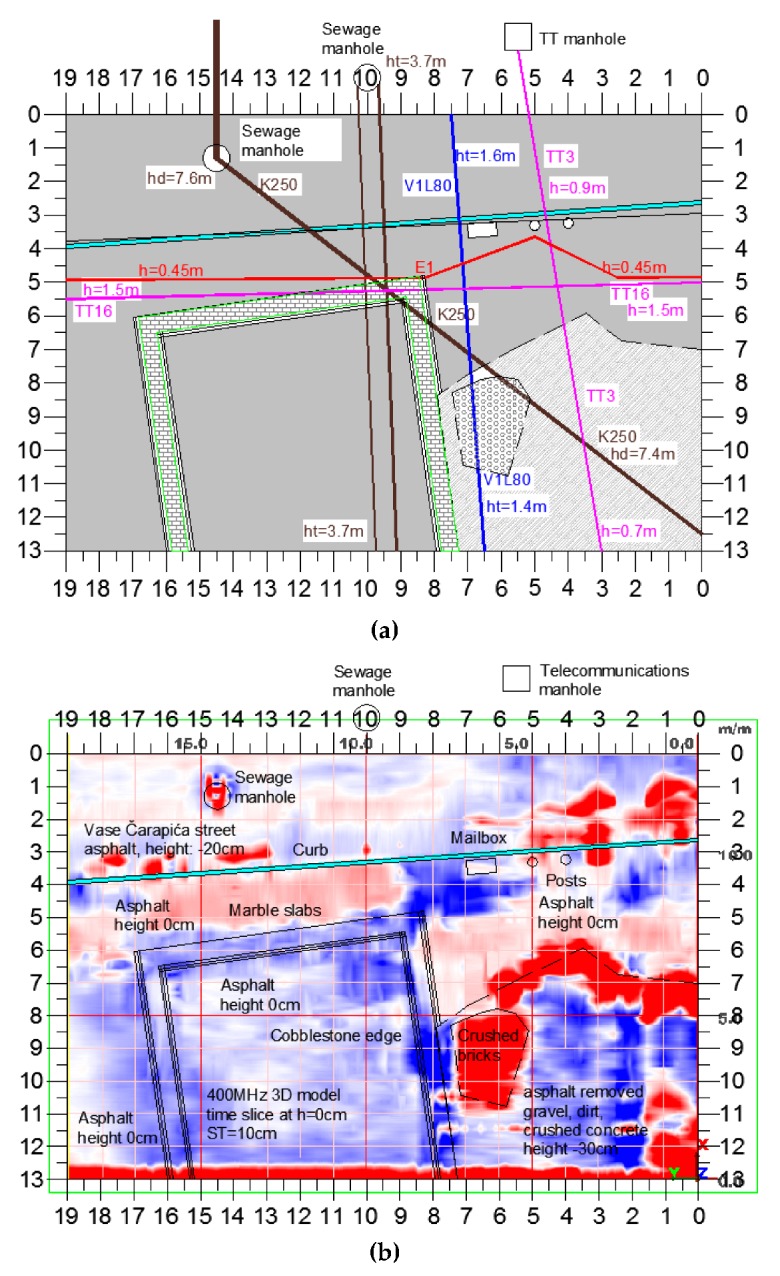
(**a**) Map of the survey area, where all the existing utilities were drawn and marked. (**b**) Ground-surface features affecting the GPR survey, overlapped with the 0-depth horizontal slice (ST = 16 cm, 400 MHz data).

To make the input data more complete and enable better data processing and interpretation, the existing utilities and their depths were marked on the ground (Figure 7). Both GPR and electromagnetic locator were used for utility detection. In particular, according to the Serbian utility cadastre, the following utilities were present:

- Sewage pipe K250: concrete pipe with Nominal Diameter DN = 250 mm, at 7 m depth;
- Telecommunication cable TT16: bunch of cables in a protective PVC tube with DN = 80 mm, at 1.5 m depth;
- Telecommunication cable TT3: bunch of cables in a protective PVC tube with DN = 80 mm, at unknown depth;
- Waterline V1L80: metal pipe with DN = 80 mm, at unknown depth;
- Powerline, at 0.6 m depth.

### 3.2. Data Editing and Processing

After the measurements, all data were edited and processed, analysed and interpreted. This work was carried out in two steps. First, all the B-Scans were considered; subsequently, the two C-Scans were created and considered. For data editing and processing, and for the creation of the 3D models, the commercial software RADAN was used, produced by GSSI Inc.

Since the fixed-offset acquisition approach was employed, correction of horizontal scale was not needed. Concerning the vertical scale, the surface was horizontal and a Time Zero Offset was applied to all radargrams, to remove the part of the signal that propagated through the air. The Position Correction value was 5.58 ns and 7.89 ns for the data collected with the 400 MHz and 200 MHz antennas, respectively.

Full pass background removal was applied to remove horizontal bands from the B-Scans. A Finite Impulse Response (FIR) bandpass vertical filter was also used, with cut-off frequencies f_HP_ = 150 MHz and f_LP_ = 650 MHz for the data collected with the 400 MHz antennas, whereas f_HP_ = 80 MHz and f_LP_ = 350 MHz for the data collected with the 200 MHz antennas.

To improve the radargram quality, an Exponential Range Gain function was applied. Gain values were specified in 10 vertical positions and the values were increasing with depth. The application of this gain improved the overall interpretability of the B-Scans and of the 3D models, by accentuating the weaker reflections, slightly reducing the strongest reflections, and by performing a vertical normalization of the profile.

**Figure 8:**
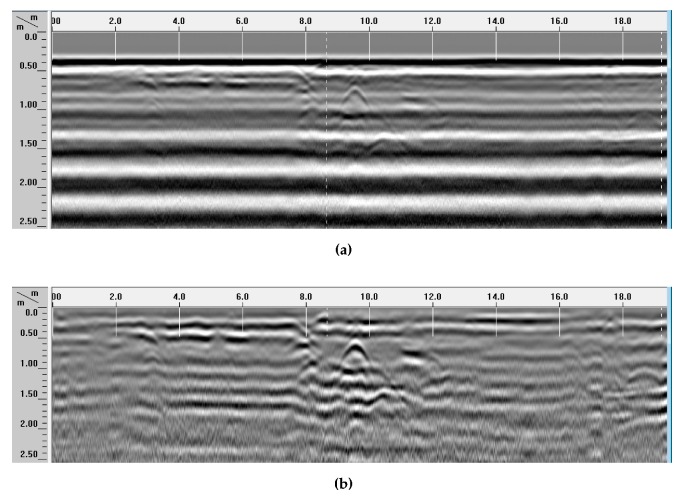
(**a**) Example of raw data collected with the 400 MHz antennas. (**b**) The same radargram as in (**a**), after the application of the editing and processing steps summarized in the text.

The same editing and processing steps were applied to all radargrams. Radargram F029 is shown as an example in Figure 8 (its location is given on the survey plan, see Figure 4).

By exploiting a procedure defined in RADAN software, for each set of antennas a 3D model was created. To create a 3D model, the processed radargrams were loaded into the software according to the layout defined in the survey plan (Figure 4).

As already mentioned in Section 3.1, when the chosen PS does not satisfy the full resolution requirements, it is necessary to apply an interpolation method to fill in the space between adjacent radargrams in the 3D model. Commercial software solutions for GPR offer the possibility to employ different interpolation techniques. Linear interpolation is applied to single direction GPR profiles and is primarily made orthogonal to the profile direction; this kind of interpolation is most often used when line-type objects are sought (walls, foundations, utilities). Bilinear interpolation is used to search for three-dimensional single targets (local features) and takes both directions of GPR profiles into computation [44]. The interpolation radius (IR) has a significant impact on the resolution and interpretability of the interpolated C-scan: a smaller IR provides more realistic results, less data smoothing and more limited loss of small features. In the case study presented in this paper, most targets were linear objects (utilities; the walls of the Württemberg-Stambol Gate) and their indicative orientation was known, therefore linear interpolation was chosen, and it was applied perpendicular to the profile direction. It is recommended that, when linear interpolation applied, 0.2 PS ≤ IR ≤ PS, hence in this case 0.1 m ≤ IR ≤ 0.5 m [44]. In this case study, IR = 0.3 m was chosen.

Horizontal slices were extracted from the C-Scans and were crucial for data interpretation. As is known, such slices show the radar reflection intensity over a certain thickness at a given depth. In particular, a single slice of a certain thickness shows in every pixel the sum of the electric-field amplitude values within the corresponding depth range. Thicker slices obviously show higher values (reflections are enhanced and more clearly visible), but they also introduce higher uncertainty regarding feature depth. For non-overlapping slices, the depth error can be assumed to be at least equal to half the slice thickness. On the other hand, with slice thicknesses much smaller than the vertical size of the sought feature, the feature cannot be fully delineated in a single slice. The use of an appropriate slice thickness (ST) is therefore especially important. A practical recommendation, for objects of unknown dimensions, is that ST should be 0.5λ ≤ ST ≤ λ [43]. According to this criterion, in the case study presented herein ST needed to be 13.25 cm ≤ ST ≤ 26.5 cm for the 400 MHz antennas and 26.5 cm ≤ ST ≤ 53 cm for the 200 MHz antennas. So, the chosen values of ST were 16 cm for the 400 MHz data and 33 cm for the 200 MHz data.

## 4. Results and Discussion

### 4.1. B-scans

Based on available information [36,37,38], the depth of the remains of the walls was unknown. The possibility that the archaeological structures could be beyond the maximum range of the 400 MHz antennas seemed realistic and for this reason data were collected using 200 MHz antennas too. However, the spatial resolution was worse with the 200 MHz antennas, hence the majority of interpretation results were based on the 400 MHz data. Nonetheless, the 200 MHz data were very useful to check the presence of structures in the region between 2 m and 5 m of depth.

**Figure 9:**
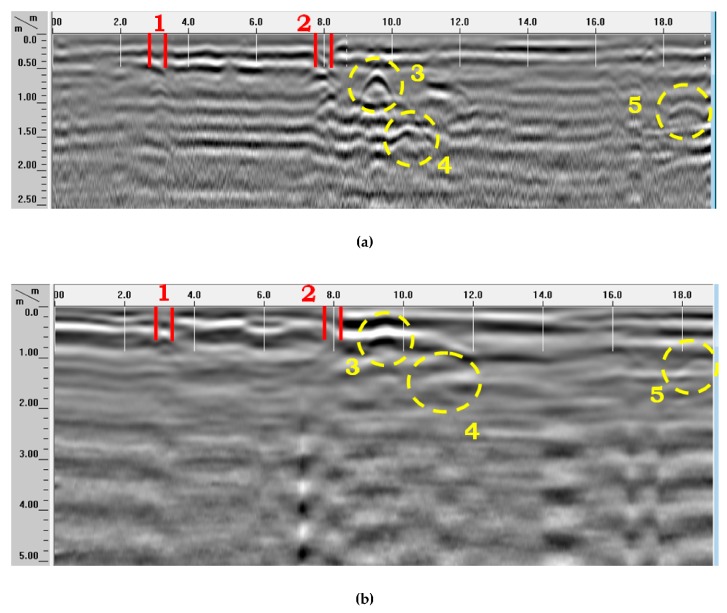
Comparative analysis example: (**a**) 400-MHz B-Scan F029 and (**b**) 200-MHz B-Scan F090; on the same trace; the signatures generated by utilities and archaeological remains are marked.

In B-Scans, several signatures were observed. Underground objects with circular cross section were detected and identified based on their hyperbolic signatures in the radargrams [49,50]. A comparative analysis of two radargrams collected along the same acquisition line by using 400 MHz and 200 MHz antennas is shown in Figure 9. In these B-Scan examples, several reflections can be seen: hyperbolas generated by circular-section utilities (indicated by 3 and 5, yellow circles); horizontal lines generated by surface layers, resulting from the antenna movement over the marble plates covering Square of the Republic (indicated by 1 and 2, red lines); and anomaly 4 (yellow circle), which looks similar to a hyperbola and is generated at the edge of the sought gate remains. The utility corresponding to anomaly 3 in Figure 9b is a waterline V1L80, buried at a depth of about 0.65 m; the utility corresponding to anomaly 5 is a telecommunication cable, buried at a depth of about 0.95 m. The anomaly generated by the edge of the gate wall is at a depth of about 1.45 m. Strong reverberation effects can be noticed in the B-Scans, too.

The penetration depth turned out to be about 5 m with the 200-MHz antennas and about 2.5 m with the 400 MHz antennas (in both cases the signal penetration was deeper than expected).

**Figure 10:**
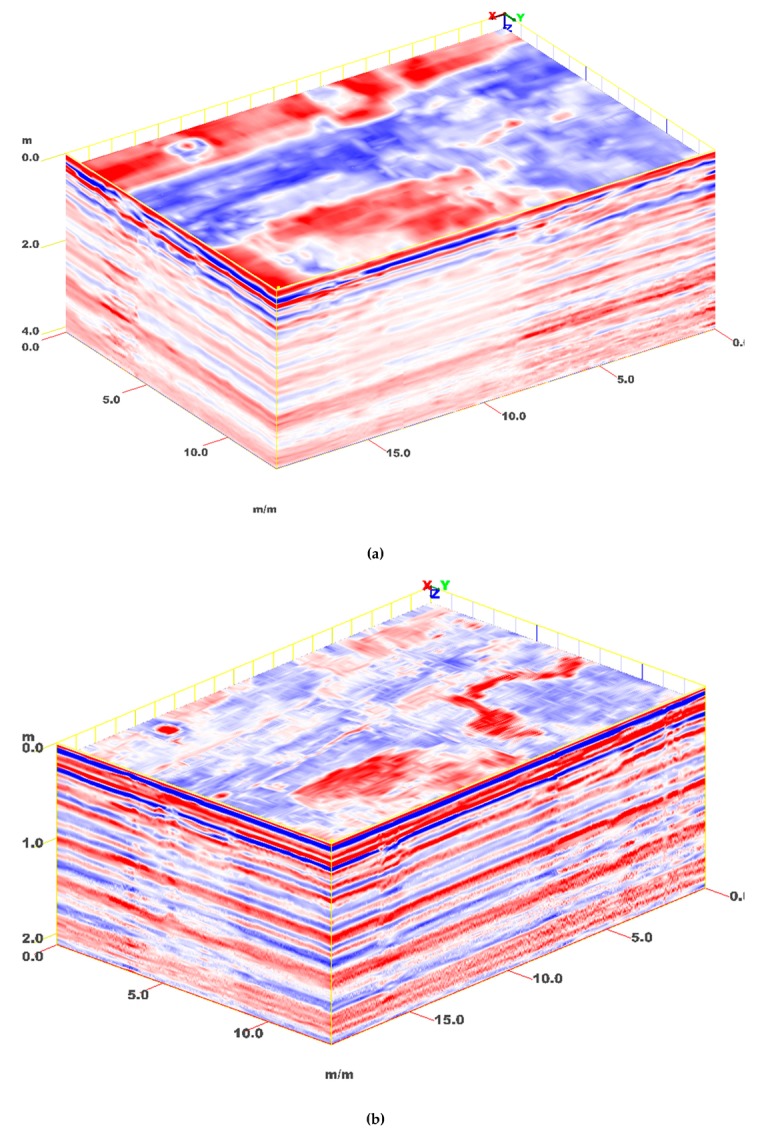
3D models, with C-Scans formed from radargrams acquired with 200-MHz (**a**) and 400-MHz (**b**) antennas.

The interpretation of B-Scans to distinguish reflections caused by underground utilities from those caused by archaeological remains is a very complex task. The analysis of radargrams F029 and F090, reported in Figure 10, as well as of further radargrams, showed that the size of utilities and remains are such that they are more clearly visible in 400 MHz radargrams. The same features can be seen on 200 MHz radargrams as well, but the quality of detection is much worse.

### 4.2. C-scans and Horizontal Slices

A more advanced analysis was based on the interpretation of C-Scans and horizontal slices. In particular, two C-Scans were created from the B-Scans recorded with the 200-MHz and 400-MHz antennas, respectively. A view of both C-Scans is presented in Figure 10. The aim of this figure is to support the Reader’s understanding of our analysis and interpretation process. Still, obviously, a two-dimensional representation (on the article’s page) of a 3D data matrix can be visually apparent and exhaustive within a certain extent, only.

Several horizontal cross-sections were extracted from both C-Scans, corresponding to different time instants and, therefore, to different depths; these slices were carefully examined, paying particular attention to reflections that could originate from the sought objects of interest. Objects that were not of a particular interest, such as buried utilities and reverberation effects, were identified in the data; the directions of utilities in the region of interest were also detected. All slices were suitably scaled and overlaid with data taken from existing documentation.

**Figure 11:**
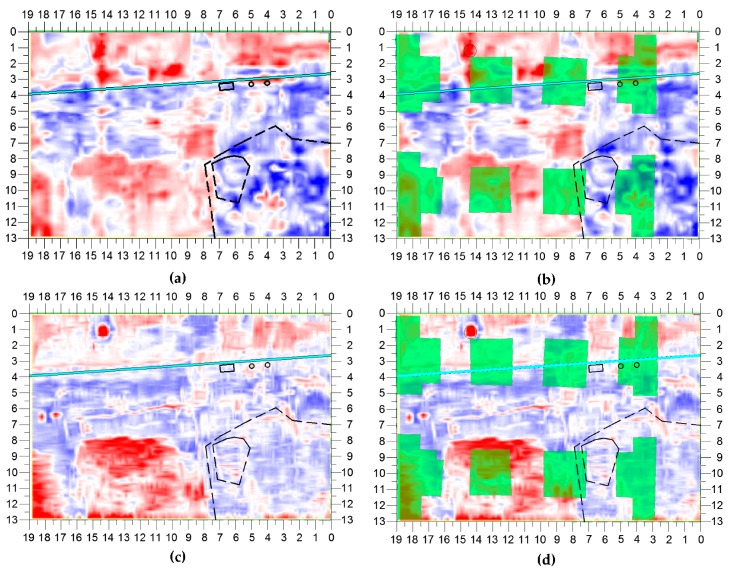
Maps (**a**) and (**b**) show the slice obtained at a depth of 80 cm from the 200-MHz data (ST = 33cm), with and without overlaid foundations in green, respectively. Analogously, maps (**c**) and (**d**) show the slice obtained at the same depth from the 400-MHz data (ST = 16 cm), with and without overlaid foundations in green, respectively.

**Figure 12:**
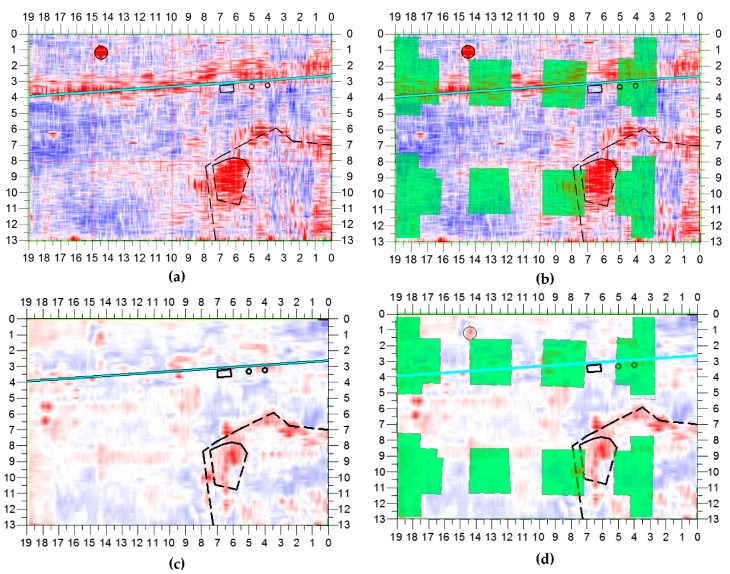
(**a**,**b**) Slice at a depth of 152 cm, with ST = 16cm, obtained from the 400-MHz data; (**c**,**d**) Slice at a depth of 330 cm, with ST = 33 cm, obtained from the 200-MHz data.

Four examples of slices are presented in Figure 11 and Figure 12, where the actual foundation remains have been overlaid to the field-amplitude maps as partially transparent green regions; on the vertical and horizontal axes of the maps, distances (in meters) are reported. The slices in Figure 11 correspond to a depth of 80 cm. Here, signatures were observed that could be ascribed to the remains of the foundations. By visually comparing the maps of Figure 11a,b with those of Figure 11c,d, the higher resolution achieved with 400-MHz antennas can be appreciated. In the lower regions of the slices, strong reflections can be seen. The reflection area in the left-bottom part of the slices coincides with the georeferenced outline of a foundation column. Another reflection area, with a few small breaks, is visible on the entire horizontal extension of both slices (from 8 m to 10 m on the vertical axis); according to the cadastre data, in this zone there are no utilities that can cause such reflections. Some results in these slices are “masked” by the surface cobble/marble plates and by the inhomogeneities of the soil; nonetheless, as many as five out of six foundation columns can be seen in the 400-MHz slice and four out of six foundation columns can be noticed in the 200-MHz slice. It was surprising to find the remains at only 80 cm of depth.

The slice presented in Figure 12a,b was obtained from data recorded with 400-MHz antennas and corresponds to a depth of 152 cm. Many reflections can be observed in this slice: one of them is generated by the manhole cover (horizontal axis: 14–15 m, vertical axis: 1–2 m); a line-shaped reflection, visible on the entire length of the slice (vertical axis: 2–4 m), is due to antenna movements over the curb. Besides these reflections, which basically are disturbances caused by scanning conditions, no other reflections representing objects of interest can be noticed. After examining all the time-slices obtained from the 400-MHz antenna data, it was actually possible to conclude that, deeper than 152 cm, no reflections caused by the foundation remains were present.

With lower-frequency antennas, the electromagnetic field emitted by the GPR penetrated deeper in the subsurface; from the data collected with the 200-MHz antennas it was therefore possible to infer information regarding deeper layers. In Figure 12c,d the cross section obtained at a depth of 330 cm from the 200-MHz C-Scan is presented: this slice matches to a high degree the one shown in Figure 12a,b. In particular, it is apparent that no reflections coming from the sought gate foundations are present.

**Figure 13:**
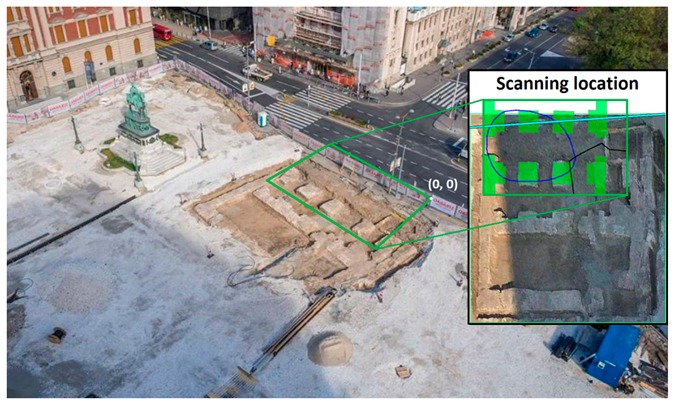
Photos of the gate foundation remains, taken after the archaeological excavation works.

Based on the results of the GPR survey, archaeological excavation works were carried out in the region where the foundation remains were estimated to be. The excavation works confirmed the presence of the foundation remains, with various columns and walls (Figure 13). It is worth noticing that the upper row of pillars, which presence was expected based on archival material, could not be detected in the GPR data. Probable reason for this is that the pillars were destroyed when the square was constructed and paved, and the material that covered the remains (most likely crumbled stone) is of very similar dielectric properties as the remains of the gate. This provides low dielectric contrast and weak reflection.

## 5. Conclusions

Ongoing urbanization and changing societies are frequently accompanied by renovation and construction works in historical cities, which may threaten important archaeological structures hidden in the subsurface. The case study presented in this paper focuses on the methodology of GPR application in archaeological prospection, with particular emphasis on the urban environment. It further corroborates that historical urban settings can strongly benefit from the use of ground penetrating radar (GPR), which is capable to map buried features within the clutter of pipes, trenches, layers, and other typical urban structures. In particular, in the case study presented herein, GPR was successfully employed to estimate the location, burial depth and geometry of the archaeological remains of the foundations of the Württemberg-Stambol Gate, in the sub-surface of the Republic Square, in Belgrade (Serbia).

The survey area was defined based on relevant historical documents. Measurements were carried out by using a commercial pulsed GPR with two sets of ground-coupled antennas having different central frequencies (200 MHz and 400 MHz). Data were recorded on a 2D grid, over a 247 m^2^ wide area, with a spacing of 0.5 m between adjacent profiles in both directions. Two 3D models were created from the measured data; several time slices were obtained from such models. The layout of the recorded profiles was identical in both cases; therefore, the models and time slices were comparable.

With the chosen spacing, it was possible to achieve an image quality that approaches the quality of a full resolution scanning (which would have been unfeasible in the Square of the Republic due to the acquisition times being too long). The parameters that mostly affect the quality of horizontal slices, in addition to the profile spacing, are the slice thickness and the interpolation method, hence these were carefully chosen. The geometrical and physical characteristics of the sought features and the presence of disturbing targets obviously have a decisive effect too: in this case study, the challenging scanning conditions caused the presence of several disturbances in the data. Nonetheless, with a careful and expert analysis and interpretation of B-Scans and slices, it was possible to clearly identify the reflections originating by the archaeological remains. All these reflections were observed at depths smaller than 150 cm and the position of the remains was estimated with very good accuracy; it was also noticed that deeper than the remains there were no significant changes on soil structures that could indicate the existence of any other interesting objects.

Based on the results of the GPR survey, an archaeological excavation was conducted in the region where the gate foundations were estimated to be, which confirmed the findings of the survey.

One of the conclusions of this study is a recommendation to incorporate GPR as a routine field procedure in construction projects involving historical cities. GPR maps and images can provide very useful information and guide the placement of excavations or define sensitive areas containing archaeological remains to be avoided for preserving and protecting our cultural heritage. In case of excavation works, from GPR images it is also possible to estimate the amount of work that will be needed to complete the excavation works and have a preliminary idea about the reconstruction possibilities.

## Figures and Tables

**Table 1:** Results of antenna footprint calculation.

Central Frequency f [MHz]	Wave Velocity v [m/ns]	Wavelength λ [m]	Horizontal/ Vertical Full Resolution Δx/Δy [m]	Antenna Footprint A [m] (1) at Depth 1 m/2 m	Antenna Footprint A [m] (2) at Depth 1 m/2 m
400	0.106	0.265	0.06625	0.40/0.73	0.37/0.52
200		0.530	0.1325	0.47/0.80	0.53/0.74
